# Longitudinal Changes in Suicide Bereavement Experiences: A Qualitative Study of Family Members over 18 Months after Loss

**DOI:** 10.3390/ijerph20043013

**Published:** 2023-02-09

**Authors:** Yan Chen, Aarno Laitila

**Affiliations:** Department of Psychology, Faculty of Education and Psychology, University of Jyväskylä, Kärki, Mattilanniemi 6, P.O. Box 35, FI-40014 Jyväskylä, Finland

**Keywords:** longitudinal changes, suicide bereavement, qualitative, family members, 18 months

## Abstract

Family members bereaved by their loved ones’ suicidal death normally undergo a complicated and lengthy bereavement process. In this qualitative case study, we explored longitudinal changes in the suicide bereavement process by applying assimilation analysis, based on the Assimilation Model (AM) and the Assimilation of Problematic Experiences Scale (APES), to longitudinal interview data collected from two Chinese suicide-bereaved individuals within the first 18 months after their loss. The results showed that over time the participants both progressed in adapting to their traumatic losses. Assimilation analysis both effectively elaborated the difference in the inner world of the bereaved and clearly demonstrated development in their adaptation to the loss. This study contributes new knowledge on the longitudinal changes in suicide bereavement experiences and demonstrates the applicability of assimilation analysis to suicide bereavement research. Professional help and resources need to be tailored and adapted to meet the changing needs of suicide-bereaved family members.

## 1. Introduction

Grief following the suicidal death of loved ones can be devastating. Cerel et al. [1] found that close family members form the majority of the bereaved who are most impacted by suicide, and that they suffer more than non-family persons from suicide-related loss. Creuzé et al. [2] also demonstrated the great impact of suicide on family members as both individuals and as a unit.

While sharing several common features (e.g., sadness) with bereavement after other types of loss, especially bereavement following unexpected decease and violent decease [3], some of these features are more evident in suicide than non-suicide bereavement. First, suicide bereavement exhibits a higher level of complicated emotional reactions and thoughts, including numbness and disbelief [4], rejection [5,6], guilt and self-blame [4], perceived responsibility [5,6], feelings of being rejected and abandoned by the deceased leading to feelings of anger and unworthiness [7], pondering on unanswered questions [8,9], and dramatic changes in one’s core belief system [10,11]. Second, individuals bereaved by suicide are at higher risk for mental health difficulties such as depression, anxiety, post-traumatic stress disorder (PTSD), complicated grief, as well as for suicide ideation, attempts and completions [11,12,13] and unpleasant situations in their social network, such as stigma and shame [5,6], embarrassment, and isolation, etc. [10,14,15]. Besides these individual-level grief reactions, various post-loss family-level changes occur, such as in the regulation of the family’s life, and in communication and interaction, mutual emotional accessibility, and cohesiveness within the family unit [11]. Creuzé et al. [2] found that family conflicts, taboos or cohesion also arise following suicidal loss.

Despite these research findings, studies on longitudinal changes in the suicide bereavement process remain scarce. Some of the few existing studies have focused on specific groups (e.g., children, parents, and older spouses) [16,17,18,19] while others have followed a quantitative approach [6,18]. Methodologically rigorous qualitative research is needed to elaborate the diverse grief trajectories of bereaved individual family members in their diverse relationships with the deceased, and to gain a clear picture of their mental status at different time points. Hence, the present study applied a qualitative case study approach, tracking the bereavement journey of two Chinese suicide-bereaved individuals over 18 months and focusing on their lived bereavement experiences at different time points. We used assimilation analysis [20,21,22], to study the data as it offers an intensive, qualitative procedure for a case study [23] and has been demonstrated to offer particular advantages for monitoring psychological changes in the processing of psychologically problematic or painful experiences [24,25,26]. 

Assimilation analysis (AA) is based on the Assimilation Model (AM) and the Assimilation of Problematic Experiences Scale (APES). The Assimilation Model (AM) is an integrative theory and framework used in accounting for psychological change processes. AA was originally applied to track changes in psychotherapeutic processes [27,28] and was later developed for studying interviews in non-therapeutic contexts [25,29,30]. This study is a further application of AA to non-therapeutic interviews conducted to assess two participants’ natural grieving status during which they received no professional intervention, although one participant participated in a suicide-bereavement support group. According to the schema formulation of AM [31], positive change occurs as the problematic experiences are gradually assimilated into one’s schemas, “schema” referring here to the frame of reference that organizes one’s perception and experience. A problematic experience can be a wish, intention, or behavior that is psychologically painful, arising from a particular life event or set of associated life events [27,32]. Coincidentally, Jordan [11] noted that suicide may disturb the presumed world or cognitive schema of bereaved individuals. 

The assimilation of individuals’ problematic experiences into their schemas is rated from zero to seven in an eight-stage process, as presented in the Assimilation of Problematic Experiences Scale (APES) [26], which contains a description of the cognitive and affective features of each stage. In stage zero (warded off/dissociated), the problematic experience is actively avoided, and accompanied with minimal affect [26]. In stage one (unwanted thoughts/active avoidance), thoughts associated with the experience arise when triggered by external circumstances, and affect becomes stronger and more salient. In stage two (vague awareness/emergence), the experience is acknowledged, the problem cannot yet be clearly formulated, and affect is acutely painful or panicky. In stage three (problem statement/clarification), the problem is clearly stated, with negative but manageable affect. In stage four (understanding/insight), the problematic experience achieves a clear connection to a schema, accompanied by both unpleasant and pleasant recognition and affect. In stage five (application/working through), understanding is used to tackle the problem, and affect is positive and optimistic. In stage six (resourcefulness/problem solution), a successful solution to the problem is worked out, with positive and satisfied affect. In stage seven (integration/mastery), solutions are successfully applied in new situations, with positive or neutral affect.

Wilson [33,34] applied AM to analyze bereavement counseling. We used assimilation analysis to analyze a single bereavement category, i.e., suicide bereavement, in specifically non-therapeutic research interviews. The research questions were:

1. What changes occur in suicide bereavement experiences over the first 18-month period after loss?
2. What are the strengths and challenges of using assimilation analysis to analyze changes in the suicide bereavement process?

Moreover, as both participants are Chinese, we explored the potential impact of Chinese culture on their grief. 

## 2. Method

### 2.1. Participants

This study constitutes part of a larger research project focusing on lived suicide bereavement experiences in China. To track the bereavement journey of suicide-bereaved individuals, focusing on their lived bereavement experiences at different time points, two participants, W and Song (both are pseudonyms) were included in this study as, at the time of their first interviews, the interval since their suicidal loss was the shortest among all the 14 participants included in the research project. They were also the only two participants in the longitudinal interviews. Specifically, W was interviewed four times at around 3, 7, 10, and 18 months after his wife (L) had died by suicide. Song was interviewed twice, at around 6 and 18 months after her younger brother (X) had died by suicide.

Both participants had received a higher education. W was over thirty. He and his late wife’s marriage was the first marriage for each, and they had no children. The marriage had lasted for 4–5 years. Song was approaching her thirties. She was the second daughter in the family and three years younger than her older sister.

### 2.2. Research Ethics

The Research Ethics Committee of University of Eastern Finland approved the study. Before the interviews, the participants were informed about the research, including the voluntary nature and anonymity of participation, the purpose and procedures of the interviews, the potential benefit and risks of participating in the interviews, their right to quit at any time, and the resources available to them if they encountered distress during and/or after the interviews. Both participants gave their written informed consent before the interviews. After the interviews, the interviewer inquired about the participants’ mental status so that timely support could be provided if needed. 

### 2.3. Procedures 

#### 2.3.1. Participant Recruitment and Data Collection

W was recruited through a suicide bereavement support group and Song through social media. The first author conducted semi-structured in-depth interviews with W and Song in quiet and private venues in China. All the interviews were conducted face to face, except for W’s third interview, which was conducted online through an audio call. The interviews focused on the participants’ bereavement experiences and process, specifically on their reactions, perceptions, and changes in these after the event, their coping and adjustment at different times, changes in their families, support sought or received, etc. The interview guide was derived from the literature on experiences and changes in suicide bereavement processes. The interviews were audio-recorded with the participants’ consent. Throughout the interviews, the interview process mostly followed the participants’ narratives. Probes and follow-up questions were proposed when appropriate. This approach enabled the interviewees to manage their narrative pace and emotions with a greater sense of control. 

#### 2.3.2. Assimilation Analysis

The first author conducted all the interviews in Chinese, transcribed the interviews verbatim in Chinese, and translated the Chinese transcript into English for analysis. A four-step assimilation analysis [26,35] previously used to analyze psychotherapy sessions was adapted and used in this study. Each step was completed with alternation between the two authors’ independent data analysis and their collaborative data sessions conducted until consensus was achieved.

Step 1: Familiarization and indexing. Through repeated listening to the audio recordings and reading the transcripts, the researchers discerned the participants’ thoughts and feelings about their loss and made a list of problematic topics. In AM, a “topic” is an attitude expressed toward an object (which can be a person, thing, event, or situation) [36]. 

Step 2: Identifying and Choosing Themes. In AM, a “theme” is an attitude revealed recurrently, possibly regarding several objects [36]. From the list of topics extracted in Step 1, themes, i.e., topics which were mentioned frequently and narrated at great length, were identified. We named every theme based on its core content. Based on their length of narration, we assigned the themes identified in each interview into three categories: focal themes, secondary themes, and tertiary themes. The focal themes were narrated at the greatest length in each interview, the secondary themes at medium length, and the tertiary themes at the shortest length. Some themes, which closely resembled each other were combined to form a single focal or secondary theme. These combined themes were named sub-themes in this study.

Step 3: Selecting Passages. Passages representing the three categories of themes were located and extracted. 

Step 4: Describing the Process of Assimilation Represented in the Passages. Each interview was assigned an overall APES rating based on the content of the themes and passages gleaned from Steps 2 and 3, respectively. We used words together with the APES ratings to elaborate our understandings of the participants’ process of assimilating their loss. 

## 3. Results

### 3.1. The Case of W

The themes identified in each of W’s four interviews are presented in Table 1 below.

#### 3.1.1. Themes and APES Ratings of Each Interview

W’s first interview has been analyzed in detail in another research article [37]. The focal themes in the first interview included *intellectualization* and *bereavement experiences. Intellectualization was* manifested in the fact that W spent most of his time talking about various scientific and philosophical topics. This theme alone accounted for 84 min of the 144-min interview. 

After being asked about the impact of his wife’s suicide on him, W vividly described his intense and overwhelming sadness and other negative emotions. He felt her death was unbelievable and sudden, and he was experiencing feelings of guilt, although these had moderated after he learned some of the reasons for her death. Moreover, it had altered his view of life, and he briefly recalled what L had been like. Fortunately, his parents’ company and his participation in the bereavement support group had helped him. W’s *perceptions of his wife’s death* were somewhat contradictory. On the one hand, W attributed L’s death to an accident while on the other, his behavior indicated that he did not reject the high possibility that L might have died by suicide. 

Owing to the frequent shifts in topics and themes, and to the great discrepancies in the APES ratings across different themes, it was not possible to give an overall APES rating of W’s first interview. However, it was agreed that W’s mental status was characterized by turbulence, contradictions, and avoidance, and hence that W’s overall assimilation of L’s suicide was still at an early stage, i.e., below 2.5 points, which is the cutoff between emergence and clarification on the APES. 

The focal themes in the second interview included *bereavement experiences and changes,* and *exploration and reconstruction of L’s suicide*. W’s narratives were less intellectualized; scientific and philosophical topics were more both briefly mentioned and more relevant to his narration of his thoughts and experiences. In the narratives under the theme *bereavement experiences and changes*, W expressed his expectation of gaining more control over his life and what he wished to achieve in the future. However, he mentioned his pain only indirectly and briefly in the second interview, his bereavement-related emotions remaining almost indiscernible.

Excerpt: 

W: Seeking truth…actually is the most important thing, sometimes, including after this thing happened, sometimes the truth of things brings a lot of pain, you need something to numb yourself, right? But you can’t numb yourself forever right?

W acknowledged L’s death as suicide, explored its possible causes, and constructed a multi-dimensional interpretation for it that involved *L’s personalities, the influence on L of her original family, L’s diaries,* and *L’s depression*. However, W’s reconstruction mainly focused on external causal explanations for L’s death and lacked self-reflection, thereby appearing incomprehensive.

W’s *self-exploration* started through reading books, pondering about his family and observing himself. He felt he had gotten past the most severe phase of guilt and self-blame and was less distressed. However, he frequently emphasized the topic of guilt and self-blame, which seemed a crucial and unavoidable part of his bereavement process. 

Excerpt:

W: She wasn’t a person like me, she didn’t get the disease because of me, after I knew that, I walked away from feelings of guilt, my conscience could quieten down …

Based on the above observations, we can see that with his more manageable emotional status, W was more accepting of his suicidal loss: he was able to confront the complexities of his bereavement, and begin his (although not yet comprehensive) exploration and reconstruction of L’s suicidal death. Hence, we rated W’s second interview as 2.8 (approaching clarification).

In the third interview, the focal themes included conflicts with ex in-laws, exploration and reconstruction of L’s suicide and bereavement experiences and changes. The largest proportion of W’s talk in the interview concerned his conflicts with his ex in-laws. He recounted in detail how L’s original family blamed him for L’s death, and the strong emotions triggered by these conflicts. 

Excerpt:

W: … So you say, rather disgusting, right? So mean, so heartless…ridiculous and bastard, really disgusting … What they did made me feel I couldn’t remain drowning in sorrow any more, I had to pull myself together to tackle those things.

On the theme of *bereavement experiences and changes*, W directly described his *current emotional status* and compared it with his *intense emotions experienced previously*. Some negative aspects emerged into W’s narration of his *memories of L*. He recognized that he was still suffering considerably from feelings of guilt and frankly elaborated the different aspects of these *feelings* caused by L’s suicide. 

Excerpt:

W: After she’d gone, I found her diaries, after seeing them, I blamed myself heavily at that time … back then, I didn’t empathize with her, she hid those things from me, I didn’t recognize them either, this made me somehow feel guilty …

Thus, it is evident that W was able to directly and openly express and manage his different emotions, including those that were negative and aggressive emotions. His reflection and reconstruction had now become more comprehensive and in-depth, and he had a clear picture of what he wanted to achieve in the future. Hence, we rated W’s third interview as 3.3 (slightly above clarification).

The focal themes in the fourth interview included *bereavement experiences and changes,* and *exploration and reconstruction of the suicide.* W relocated to live alone at some point between the third and fourth interview. He said that emotionally he could “come to terms with reality”, i.e., his wife’s suicide. He had also taken action on his future. W summarized some of the things that had helped his recovery, including his hobbies, his zest for reading and thinking, his friends, the bereavement support group, etc. He remembered what L was like and thought about her personalities dialectically. 

Excerpt:

W: Truth is the thing that you must come to terms with, it is just sometimes too cruel to be accepted…You are just a minute star in the universe, you have such a short life, we are so minimal, this thing is not a big deal…

On the theme *exploration/reconstruction of the suicide,* W newly added that L probably felt great pressure in their marriage due to her physical illness. *Conflicts with parents* accounted for a greater proportion of his talk in the fourth than previous interviews. W recounted the difference in ideas and habits of living between himself and his parents. He assessed his current feelings of guilt as “appropriate”.

Excerpt:

W: I read her diaries, and then I acquired some knowledge about psychology, I got to know what was going on, I didn’t blame myself too much, actually a certain amount of guilt is unavoidable, I think it is appropriate guilt, not too much …

Thus, it can be seen that by the time of his fourth interview, W had taken actions to achieve what he wanted and had become more flexible and proficient at expressing and regulating his emotions, more aware of what was helpful and unhelpful for recovering from his bereavement. We rated W’s fourth interview as 3.7 (approaching insight).

#### 3.1.2. Development of Bereavement across the Four Interviews

W’s changes in bereavement are shown in the development of themes across the four interviews. W’s talk in the later three interviews was much more stable than in the first interview, which was characterized by frequent switches between themes and fluctuation in their APES ratings. Moreover, the themes identified in the four interviews underwent various changes, either existing in only one or two interviews, evolving into other themes, or increasing or decreasing in the length and depth of their narrative content (see Table 1). 

The theme *bereavement experiences and changes* was the only focal theme common to all four interviews. However, its position and sub-themes in the interviews varied. In regard to the contained sub-themes, in the first interview, no “changes” were included; in the second interview, *seeking truth* and *future expectation* emerged, however, expression of emotions was almost hidden. In the third interview, emotions were expressed, and he went into more detail in *future expectations*. In the fourth interview, W’s narratives on this theme were more down to earth. 

The theme *exploration and reconstruction of L’s suicide* was the second focal theme in the remaining three interviews. While containing the same sub-themes as in the second and third interview, the underlying narrative content underneath of each sub-theme was much more detailed and profounder in the third interview. The fourth interview contained a new sub-theme, *L’s worry, and feelings of pressure in the marriage*.

*Feelings of guilt* were also present across W’s four interviews. In the first interview, it was a sub-theme. He was experiencing guilt about L’s death, although this had moderated once he had learned some of its causes. In the later interviews, it was a secondary theme. In the second interview, W talked at length on this theme, despite saying that he had already left behind his most severe feelings of guilt. In the third interview, W was still burdened by guilt. In the fourth interview, however, W concluded that his feelings of guilt had moderated to an appropriate level.

### 3.2. The Case of Song

#### 3.2.1. Themes and APES Rating of Each Interview

The themes identified in each of Song’s two interviews are presented in the table below. 

The focal themes in the first interview were *exploration and reconstruction of the suicide*, *emotions caused by the suicide* and *impact of the suicide*. Song talked about her brother’s suicide candidly. She sought to understand his inner world and the reasons or even THE reason for it, possible advance warnings, and to figure out what family-related issues might have impacted his choice of suicide. The suicide has also caused her to feel a range of intense *emotions* (see Table 2). 

Excerpt:

Song: In fact, quite often, until now, I feel this thing is too unreal, difficult to accept, I can’t figure it out…If I could have brought him into my life, then maybe he wouldn’t have done this, but maybe it’s useless…I am not surprised he had these thoughts, I often had these thoughts when I was a child… This is irreversible, I feel I have been totally changed, my whole life…Such pain can’t be measured, and there is no solution…

While Song was clear about the *impact of the suicide* on her and her family, she was “puzzled” about what areas she could work on and how to cope with her intense emotions in practice. Her current life arrangements and future plans had changed. She spent a lot of energy caring for her parents and had also had to postpone making decisions on her career development and romantic relationship due to her “too tired/exhausted” state. 

We rated Song’s assimilation of her brother’s suicide on the APES as 2.7.

Song’s second interview lasted 283 min. The largest proportion of her talk was spent giving a detailed description of her tangled romantic relationship with her boyfriend. Her quarrels with him had become more serious since X’s suicide. 

Song: If he’s so close to his cousin, why can’t he understand my affection for my brother, I’m very angry about this, thinking why can’t you understand the trauma in my heart…Well, it may be, I think this kind of trauma may be caused by my brother, it may be…

Int: How about before? Were you like this before the event?

Song: Before, it wasn’t so serious before.

In her secondary theme, *bereavement experiences and changes in family members*, Song’s life seemed to have moved on, since her focus had shifted away from caring for her parents to implementing her career plan. Nevertheless, Song manifested ambivalence at several points. Cognitively, she could understand why her brother had died by suicide but was emotionally unable to accept it. The deliberate nature of suicide challenged her belief about the controllability of the world and life, making her feel both angry and resigned. She tried to prevent her thoughts and emotions about X’s suicide affecting her daily life. Paradoxically, in the interview, she recalled the method and scene of his suicide and her family’s bereavement experiences immediately afterwards in vivid detail, as if the event had happened only a few days ago.

Song: Now as soon as I think about the details, I’ll definitely be overwhelmed immediately, I force myself not to think about it, I need to move forward … Now my dad basically doesn’t mention it anymore, and my mom doesn’t either, she won’t mention my brother constantly like before, she also just wants to forget it.

*The impact of the family environment, family history and family relationships on Song* was a secondary theme. Here, Song’s attention focused on how her family influenced her, including her relationship with other family members and her choices in her romantic relationship.

In this interview, several narratives were intertwined, and Song freely switched between them. The same story line was scattered across the interview. Song seemed to give a comprehensive and accurate introduction to her life, covering every single aspect from past to present. The main story line among the many concerned Song’s *tangled romantic relationship* with her boyfriend, with her brother’s suicide and the family’s bereavement forming an implicit and underlying motif throughout the interview. This may coincide with Song’s stated choice of not thinking about X’s suicide. 

Song’s assimilation of her brother’s suicide was rated 3.2 on the APES.

#### 3.2.2. Development of Bereavement across the Two Interviews

Song’s first interview focused on her *exploration and reconstruction of the suicide*. She also spent much time in the interview reflecting on the *various emotions X’s suicide* aroused in her and on the *impact of his suicide* on the entire family. In comparison, the focus in the second interview had shifted onto her *tangled romantic relationship* and her family’s influence on her life. Moreover, in the first interview, Song narrated her various emotions at great length, while in the second interview, her emotions were less evident. 

### 3.3. Comparison of the Two Cases

#### 3.3.1. Commonalities

Both W and Song had a comparatively high level of psychological mindedness, meaning that they were aware of their own psychological processes and could elaborate these in clear and rich language. They were also eager to integrate the psychological knowledge they had acquired into their interpretation of their close ones’ suicides. Moreover, their religious interest or belief eased their grief to some extent.

The APES ratings of both participants’ assimilation of their loss increased over time. W and Song both displayed emotions more in the earlier interviews. These emotions were characterized by ambivalence, turbulence, fluctuation, and detachment. Later, their emotions were less apparent. Moreover, their interview themes were interconnected. For example, the focal theme *bereavement experiences and changes* and *exploration/reconstruction of the suicide* impacted on each other. Lastly, Song’s first and W’s second interview occurred at similar interval after their loss, at 6 and 7 months, respectively. Coincidentally, their APES ratings of 2.7 and 2.8, respectively, were also similar. 

W and Song also shared some family-level commonalities. The families of both participants played an important role in their bereavement. W’s parents supported him with their presence while Song greatly supported her parents. Her relationship with her parents and sister also impacted her bereavement process. Moreover, both families had more intra-familial communication and interaction post- than pre-suicide, although the conflicts between family members escalated. 

#### 3.3.2. Differences

Compared to W, Song’s emotions were more explicit, diversified, and more frequently observed, especially in her first interview. Song attributed her brother’s death to suicide from the very beginning whereas W had doubts about the nature of his wife’s death. Moreover, probably owing to their different relationship to the deceased, Song’s account included more about other family members’ bereavement experiences.

Both W and Song’s last interview took place at 18 months after their loss. At the time of the last interview, W had drawn on his inner and outer resources to create a channel for his grief and recovery. He had arrived at a balanced and peaceful phase of grieving after having undergone turmoil, distress, and conflict with his ex in-laws. In comparison, Song continued to experience difficulty in dealing with the overwhelming emotions aroused by thinking about X’s suicide. Dramatic and conflictual voices filled her inner world, causing her to be more avoidant when coping with her bereavement. The difference in W’s and Song’s status at their last interviews is reflected in their APES rating: 3.7 (W) and 3.2 (Song). 

## 4. Discussion

Bereavement occasioned by suicide is normally a complicated and long process. The mental status of bereaved individuals varies at different time points after their loss. W and Song both experienced changes and progress in their bereavement during the first 18 months after loss. W journeyed from suffering overwhelming, detached and turbulent emotions, and experiencing a considerable void in his heart and life, to constructing causes for his wife’s suicide from different perspectives, dealing with the conflicts triggered by his loss, confronting negative emotions, and finally arriving at a balanced and peaceful phase of grieving. While Song also started from being overwhelmed by intense emotions, she ended up experiencing dramatic mental conflicts and intentionally avoiding mention of her loss.

The grief trajectories of W and Song support the findings of Gaffney and Hannigan [38] on the initial, medium-term and long-term stages of coping with grief. Dealing with complicated emotions is an essential part of suicide bereavement experiences, as the present two cases show. The intense emotions revealed by the participants in their initial interviews are a previously reported feature of bereavement reactions in the months immediately following a suicide [39,40]. W’s emotions were detached at 3 months and hidden at 7 months post loss. Song, in turn, displayed obvious avoidance at 18 months post loss. Ross et al. [19] considered avoidance a maladaptive strategy at 6 and 12 months after suicidal loss. However, views vary. For example, Gaffney and Hannigan [38] found avoidance to be a regulatory strategy, Wilson [34] suggests that detachment and avoidance may facilitate temporary respite from intense grief, while Updegaff and Taylor [41] suggest that avoidant coping can be helpful temporarily.

Along with the expression and regulation of emotions, exploration/reconstruction of the suicide, i.e., sense-making and meaning-making of the suicide, have been demonstrated to be a crucial stage in suicide bereavement. Sands and Tennant [42] posited that reconstruction can help bereaved persons progress in their bereavement trajectory. The significance of exploration/reconstruction for suicide bereavement has also been empirically supported [8,19,38,39,43,44].

In line with Shields, Kavanagh, and Russo [44], who found that the three main themes underlying the process of bereavement, i.e., the feelings of bereavement, the meaning of bereavement, and the context of bereavement, may have a large impact on one another, the themes in the present participants’ interviews were interconnected. Studies have suggested that reconstruction of the suicide story can help the bereaved bond with the lost family member in a more positive way, lessening their sense of guilt [42,45]. In our study, the two most prominent themes—bereavement experiences/emotions caused by the suicide and exploration/reconstruction of the suicide—were interrelated and affected each other’s development.

Assimilation analysis effectively elaborated the differences in the participants’ inner worlds and clearly demonstrated their adaptation to their loss over time. The extraction of themes and related passages from the transcripts showed the prominence and valence of each theme, indicating their sequence in the process of suicide bereavement and giving a clear picture of the participants’ real-time grieving status. Comparison of the APES ratings and thematic content across the different interviews clearly revealed the changes in the participants’ suicide bereavement process. Thus, the application of assimilation analysis in this study rendered visible not only the micro details in the different phases of bereavement experiences, but also the underlying macro changes over time. This could hardly have been achieved with the research methods used in previous studies on suicide-bereaved individuals’ grief trajectories [6,17,18,19,46].

We conducted in-depth individual interviews with the two participants. Research has shown that such interviews can have an interventive impact on participants, even if unintended [47]. Bonanno, Boerner, and Wortman [48] found that talking about a deceased spouse was beneficial for resilient individuals. Similarly, Baddeley and Singer [49] suggest that the bereaved can make meaning of their bereavement by disclosing their grieving experiences to other people. Shields, Kavanagh, and Russo [44] propose that the act of creating an understanding and non-judgemental environment that allows the bereaved to communicate their experiences candidly and honestly can help them through their grieving process. Here, W was interviewed at a higher frequency and shorter interval than Song. The potential interventive impact of W’s four interviews and/or his participation in a bereavement support group may partly explain his better final status. Research has confirmed the positive function of bereavement support groups [19,38,50]. Participation in research interviews and in support groups provides opportunities for the bereaved to talk about their grieving experiences with others and potentially find meaning in their bereavement.

Ali [51] suggests that consideration of the indigenous cultural context is crucial for generating knowledge on adaptive reactions to grief. The two present cases shed light on the impact of Chinese culture on individuals’ bereavement. The intensity of the participants’ feelings of guilt and self-blame stem partially from their sense of failing their responsibilities as a husband and as an older sibling. This reveals the uneven distribution of responsibility and the hierarchy in family relationships in Chinese culture. For Song, caring for her parents became her most important bereavement-related task during the first year after the event, as she had to be strong for her family. Chinese families widely value traditional filial piety. This factor may have informed Song’s strong sense of responsibility towards her parents and her blaming of her deceased brother, as suicide is deemed as extremely unfilial act in Chinese culture [52]. Hence, Song’s family also experienced awkwardness in their social network after their loss, an added burden, especially at the onset of their bereavement. Research has also shown that, particularly in Asian cultures, stigma associated with mental illness casts a shadow not only over the affected individuals but often also over their families [53,54].

### 4.1. Strengths and Limitations

A strength of this study is that it is one of the few to monitor suicide bereavement trajectories over a longer period. Utilizing in-depth interviews, the study tracked the two participants over 18 months, thereby amassing rich and detailed longitudinal data on their experiences. These factors, together with the application of assimilation analysis, enabled the main features of the bereaved individuals’ inner worlds to be charted at different times, revealing how they adapted to their loss. Hence, our study extended the (thus far) limited knowledge on changes in suicide bereavement experiences over time, while also demonstrating the applicability of assimilation analysis to this research domain. 

We also applied various methods to guarantee the trustworthiness of this study. Many of these methods enabled us to meet the qualitative research criteria suggested by Lincoln and Guba [55], Creswell and Miller [56] and Korstjens and Moser [57]. The methods included prolonged engagement (thorough preparation of the data collection phase; allowing sufficient time to gain familiarity and create a relationship of trust with the suicide-bereaved participants; adequate interview length; a long time span between successive interviews), methodological triangulation (complementing the in-depth interviews with field notes to provide reference points for the data analysis), investigator triangulation (close collaboration between two researchers; alternation between the authors’ independent data analysis and their collaborative data sessions), persistent observation (going back and forth between the dataset and data analysis; reading relevant theorical and empirical literature throughout the research process; allowing observations that emerged from the data to prompt ideas about the data analysis while also allowing the data analysis impact the subsequent data collection; the data analysis started immediately after each interview and continued until the article was finalized), transferability (giving a rich account of the research process and context, including the participants as well as the research data), dependability and confirmability (detailed descriptions of the analyses and interpretations made and derived from the data), reflexivity (the first author kept a research journal to keep track of her ideas and thoughts in all phases of the research so that she could reflect on her own role in each phase and, if necessary, make self-corrections).

Since, according to Levitt [58], “qualitative generalization” refers to the phenomenon rather than the population, the findings of this research can to some extent, depending on the context of the bereavement and characteristics of the bereaved, be generalized to the suicide bereavement process and the longitudinal changes that occur during it. However, it should be noted that for several reasons, generalizing from this research to wider populations is limited. First, the number of cases and interviews was small. Second, the difficulty of finding participants who had recently lost loved ones to suicide and were willing to participate in longitudinal interviews raises the possibility of selection bias. Third, the level of psychological mindedness and understanding of psychological knowledge of the present participants is not commonly encountered in the field. Finally, owing to resource constraints, we could not extend the longitudinal interviews beyond 18 months after loss. Thus, it is possible that a longer time span might better facilitate comparative research on this topic.

### 4.2. Clinical Implications

The trajectories found in this study may be of value to those who help people bereaved by suicide, including health professionals, social workers, volunteers, family members, and friends. Forms of assimilation analysis can be applied in in-depth assessment interviews with bereaved individuals to understand their adaptive processes. Our results indicate that professionals should bear in mind that the mental status of persons bereaved by suicide differs both between and within individuals over time. Hence, professional interventions and other social resources targeted to bereaved family members must consider their specific situations and tailor support to meet their changing needs. For bereaved persons suffering from long-term emotional dysregulation and severe or chronic stress symptoms such as anxiety or depression, professionals should evaluate and monitor their risk for developing complicated grief, PTSD, or suicidal tendencies, etc. Finally, coordinated culturally appropriate assistance and services can help promote the recovery of family members.

## 5. Conclusions

This study tracked the bereavement journey of two suicide-bereaved individuals and their lived experiences of bereavement at different time points during the first 18 months after loss. Although the mental status of these individuals varied both intra- and inter-individually over time, both underwent a complicated and lengthy process, showing a positive trend towards recovery from their traumatic loss. This study also demonstrated the applicability of assimilation analysis to research on changes in suicide bereavement experiences over time. We further found that participation in a bereavement support group and in individual in-depth research interviews seemed to have a positive effect on these suicide-bereaved individuals. We also speculated on the possible impact of the Chinese culture on suicide bereavement in these two cases. The findings of this study can contribute when designing more appropriate measures for helping bereaved individuals varying in the characteristics of their bereavement process.

## Figures and Tables

**Table 1:** Themes in each of W’s four interviews.

	Themes	APES Rating
First interview (3 months after his loss) Length in minutes: 143:26	**1 Intellectualization** **2 Bereavement experiences** (emotional reactions; incredibility of L’s death; feelings of guilt; what helps bereavement; view of life; memories of L) ***Perception of L’s death*** (how L died; L’s depression) Self-observation	Not assessed
Second interview (7 months after his loss) Length in minutes: 119:42	**1 Bereavement experiences and changes** (future expectations; seeking truth; what helps bereavement; memories of L) **2 Exploration/reconstruction of the suicide** (L’s personalities; influence on L of her original family; L’s diaries; L’s depression) ***Feelings of guilt*** ***Self-exploration*** Conflicts with parents Suicide prevention	2.8
Third interview (10–11 months after his loss) Length in minutes: 230:50	**1 Conflicts with ex in-laws** (details of the conflicts; L’s original family blamed W for L’s death; emotional reactions triggered by conflicts) **2 Exploration/reconstruction of the suicide** (L’s personalities; influence on L of her original family; L’s diaries; L’s depression) **3 Bereavement experiences and changes** (Current mindset; incredibility of L’s death; description of the intense emotions experienced previously; future expectations; what helps bereavement; impact of conflicts’ on bereavement) ***Memories of L*** ***Feelings of guilt*** ***View of life*** Self-exploration Conflicts with parents Few people to talk to about the suicide	3.3
Fourth interview (18 months after the loss) Length in minutes: 147:29	**1 Bereavement experiences and changes** (changes in living arrangements; previous emotional status and current emotional status; status of daily life; acceptance of truth/reality; what helps bereavement; view of life; memories of L) **2 Exploration/reconstruction of the suicide** (L’s worry and pressure during marriage; influence on L of her original family; L’s diaries; L’s depression) ***Conflicts with ex in-laws*** (details of conflicts; emotional reactions triggered by conflicts) ***Feelings of guilt*** ***Conflicts with parents*** Few people to talk to about the suicide	3.7

Note. Bold: focal themes; bold and italics: secondary themes; normal: tertiary themes; normal in parentheses: sub-themes. Numbers mark the sequence of the focal themes. L is the pseudonym used to refer to W’s late wife.

**Table 2:** Themes included in each of Song’s two interviews.

First (6 Months after Her Loss; 94 min)	Second (18 Months after Her Loss; 283 min)
**1 Exploration and reconstruction of the suicide** (family environment, family history and family relationship related to the suicide; ignored advance warnings of the suicide; extended family’s experiences of depression and attempted suicide; parental education; personalities and personal experiences of X; reflection on Song’s personal experiences) **2 Emotions caused by X’s suicide** (incredibility of X’s suicide; unable to understand or accept it; unable to have done something to prevent it; partial understanding; unchangeable and overwhelming; inestimable and unresolvable pain; pity and tragedy; loss of interest; blame/hate; compassion; feelings of guilt; dazed/resigned) **3 Impact of the suicide** (impact on socializing; changes in living arrangement; different impact of X’s suicide on different family members; impact on family relationship; impact on career plan and romantic relationship; impact on the extended family; description of suicide method and scene) View of life Religious belief Few people to talk to about X’s suicide Suicide prevention	**Tangled romantic relationship** (conflicts in relationship; consideration of relationship and marriage; status of the relationship; boyfriend’s family background; boyfriend’s relationship history and marriage; disliked marital status) ***Bereavement experiences and changes in family members*** (changes in living arrangements; impact of X’s suicide on socializing; different impact of X’s suicide on different family members; changes in view of life; carrying out career plan; emotional status; difficulty of emotionally accepting X’s suicide; ambivalence between understanding and misunderstanding of X’s suicide; description of the suicide method and scene and bereavement experiences immediately after X’s suicide; similar suicide bereavement experiences to someone else’s) ***Impact of family environment, family history and family relationship on Song*** (family relationship after X’s suicide; conflicts with sister and mother; sister’s marriage situation and brother-in-law’s family background) Exploration and reconstruction of X’s suicide (family environment, personalities, personal experiences; triggers and advance warnings of his suicide; suicide note) Disliked living status Religious belief Song’s personal experiences and personalities Career development Best friends/flatmates and friends Suicide prevention
Overall APES rating: 2.7	Overall APES rating: 3.2

Note. Bold: focal themes; bold and italics: secondary themes; normal: tertiary themes; normal in parentheses: sub-themes. X is the pseudonym used to refer to Song’s late brother.

## Data Availability

The data are not publicly available due to their confidential nature.
